# Building on Synthetic Immunology and T Cell Engineering: A Brief Journey Through the History of Chimeric Antigen Receptors

**DOI:** 10.1089/hum.2021.165

**Published:** 2021-10-18

**Authors:** Hinrich Abken

**Affiliations:** Department of Genetic Immunotherapy, Regensburg Center for Interventional Immunology (RCI), Regensburg, Germany.

**Keywords:** T-body, immunoreceptor, CAR, chimeric antigen receptor, TCR, T cell receptor, adoptive cell therapy

## Abstract

Advancement in our understanding of immune cell recognition and emerging cellular engineering technologies during the last decades made active manipulation of the T cell response possible. Synthetic immunology is providing us with an expanding set of composite receptor molecules capable to reprogram immune cell function in a predefined fashion. Since the first prototypes in the late 1980s, the design of chimeric antigen receptors (CARs; T-bodies, immunoreceptors), has followed a clear line of stepwise improvements from antigen-redirected targeting to designed “living factories” delivering transgenic products on demand. Building on basic research and creative clinical exploration, CAR T cell therapy has been achieving spectacular success in the treatment of hematologic malignancies, now beginning to improve the outcome of cancer patients. In this study, we briefly review the history of CARs and outline how the progress in the basic understanding of T cell recognition and of cell engineering technologies made novel therapies possible.

## Introduction

Historically, treatment regimens in oncology are based on three pillars, namely, surgery, chemotherapy, and radiotherapy. Advancement in the basic understanding of the molecular processes involved in T cell recognition and the application of synthetic immunology technologies made it possible to reprogram patients' own immune cells to recognize and combat cancer. These developments during the last decades are providing the basis of rationally designed immunotherapy that is now recognized as the fourth pillar in cancer therapy.

The recent breakthrough in cancer treatment with designed immune cells is a result of a long development in biomedical research and in cellular engineering technologies reaching back to the early times of cellular immunology. It is more than a century ago when Paul Ehrlich presented the hypothesis that the immune system has the power to selectively attack and control cancer.^1^ Only modern molecular immunology, genetic engineering, and cell manufacturing technologies provided us with tools to test for the hypothesis and to establish immuno-oncology in clinical practice. Within this development, it is just three decades ago that the first synthetic immune receptors were genetically engineered to engraft predefined T cell specificity and to redirect immune effector functions. Among these, chimeric antigen receptor (CAR)-redirected T cells were crowned with success when entering clinical exploration, becoming the first commercial gene transfer therapy being approved by the U.S. Food and Drug Administration (FDA) in August 2017. The FDA Commissioner at that time, Scott Gottlieb, noted the significance of the approval stating that the CAR T cells represent a “milestone in the development of a whole new scientific paradigm for the treatment of serious diseases.”^2^ While still away from being perfect, CAR T cell therapy provides hope for patients whose cancer became resistant to traditional therapies.

In this study, we briefly outline the major steps in the continuous evolution of the CAR design from the perspective of synthetic immunology and focus on redirecting engineered T cells against cancer. The review aims to indicate how the progress in both the understanding of molecular processes in T cell recognition and immune cell engineering technologies made novel therapies possible that are redirecting patient's own immune cells to attack cancer.

## The Basis of Adoptive Cell Therapy: T Cells are Capable to Control Cancer

**Figure 1.**
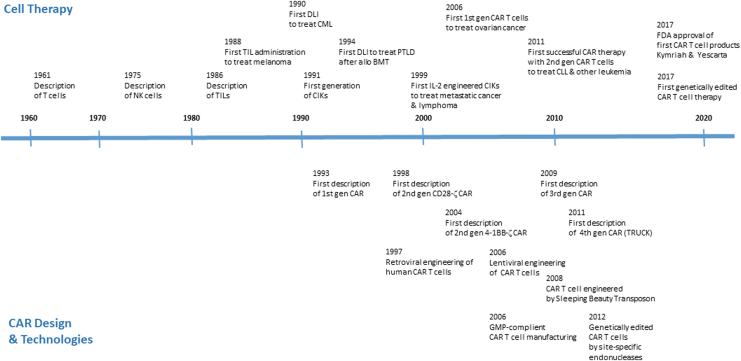
Overview of major steps in the development of adoptive T cell therapy.

Since the discovery of T cells originating from the thymus by Miller in the early 1960s^3^ and the discovery of their lytic activities, cytotoxic T lymphocytes (CTLs) were recognized as major effectors that initiate and mediate an antigen-specific lytic immune response against infected cells and cancer (Fig. 1).^4^ On the other hand, natural killer (NK) cells, first described by Kiessling *et al.* (Karolinska Institute, Stockholm) in 1975,^5–8^ were identified to kill target cells in an antigen-independent fashion, which laid the basis to understand the innate immune response in the control of cancer.

Aiming at using the lytic power of the cellular immune response, Rosenberg and his team at the NCI (Surgical Branch, NIH, Bethesda, MD) established technologies in the early 1980s to generate *ex vivo* cytotoxic cells in the presence of interleukin (IL)-2, so-called lymphokine-activated killer (LAK) cells, an enriched cell product with lytic activities.^9^ LAK cells attracted interest due to their ability to kill cancer cells *in vitro* that are resistant to NK cell-mediated lysis. These efforts resulted in 1985 in the first safe administration of LAK cells together with IL-2 to patients with metastatic cancer.^10^ In subsequent trials, however, the benefit of LAK cells proved small in comparison to IL-2 administration^11,12^ making research in more antigen-specific cytotoxic immune cells necessary.

In contrast to NK cells, T cells recognize targets in an antigen-specific fashion and, once activated, can eliminate cancer cells by various means, including perforin/granzymes, Fas ligand, and cytokines like interferon (IFN)-γ and tumor necrosis factor (TNF)-α; however, their activation requires presentation of the respective antigen by professional antigen-presenting cells (APCs). In this situation, melanoma became a tumor of particular interest to study due to its high T cell immunogenicity.^13^ An additional advantage of studying melanoma at that time was that patient-specific tumor lines could be established from surgical specimens and infiltrating immune cells, in particular tumor infiltrating lymphocytes (TILs), could be isolated and propagated *in vitro*.^14^ These T cells recognizing autologous cancer cells appeared to be much more prevalent in melanoma patients for reasons not understood at the time. Melanoma cells frequently undergo high mutation rates displaying mutated peptides as altered self-antigens on the cell surface, where they can be recognized by T cells.^15,16^

Moreover, there is an intense communication network between innate and adoptive immunity within the tumor lesion, in that also cancer cells finally participate to escape destruction. These observations made clear that cancer patients' immune system is actively experiencing tumor cells,^17,18^ mostly for long periods of time, and accumulating T cells within the tumor correlate with prognosis.^19^

By putting these observations together, tumor resident T cells were disputed to transfer antitumor immunity. It was until 1986, when the hypothesis was experimentally evaluated by Rosenberg *et al.* (NCI, NIH) by isolating TILs from melanoma lesions and amplifying the cells in the presence of recombinant IL-2 for several weeks.^14^ The *ex vivo* amplified TILs recognize melanoma-associated antigens and lyse autologous tumor cells in an *in vitro* short-term assay.^20^ A milestone toward specific T cell therapy of cancer was made in 1991 when van der Bruggen *et al.* in Boon *et al.*'s team (Ludwig Institute for Cancer Research, Brussels, Belgium) identified the antigen recognized by a tumor-reactive T cell clone from a melanoma patient.^21^ Many other tissue differentiation antigens shared by melanomas and melanocytes were subsequently cloned by a similar procedure.^22,23^

Built on these laboratory observations, amplified autologous TILs were applied together with IL-2 to melanoma patients producing substantial tumor regression, in some cases for several months and years. The pioneering work first established that systemic infusion of tumor-isolated, *ex vivo* amplified, and activated autologous T cells can induce an antitumor response.^24^ Together with the stepwise refinement of the clinical protocol, including the introduction of transient nonmyeloablative lymphodepletion before T cell transfer, called host preconditioning (reviewed in reference^25^), the Rosenberg team paved the way for clinical application of adoptive T cell therapy in general and specifically for the treatment of melanoma. The TIL approach, unfortunately, was not applicable to all melanoma patients and not to most other malignancies, indicating the need to engraft tumor specificity to naive T cells from patients' peripheral blood that has the advantage that these cells can more easily be obtained and *ex vivo* amplified for adoptive transfer.

Two years after Rosenberg's report on a TIL trial, Kolb *et al.* (Technische Universität München, München, Germany) demonstrated therapeutic efficacy of allogeneic donor lymphocyte infusion (DLI) in combination with IFN-α for the treatment of recurrent chronic myeloid leukemia^26^ in patients after allogeneic stem cell transplantation (allo-SCT). In 1994, O'Reilly achieved by DLI regression of Epstein-Barr virus (EBV)-associated posttransplant lymphoproliferative disease after allo-SCT.^27^ The pioneering work by these groups together laid the basis of the concept that adoptive transfer of *ex vivo* amplified T cells is capable to control cancer.

The elucidation of the heterodimeric structure of the T cell receptor complex (TCR) for antigen recognition^28–30^ and the development of efficient gene transfer technologies paved the way to genetically engineer T cells with antigen specificity. In a significant step forward toward antigen-specific T cell therapy, human T cells were equipped *in vitro* by gene transfer technologies with transgenic TCR αβ chains gaining human leukocyte antigen (HLA)-restricted specificity for a cancer-associated antigen.^31,32^ In that time, research was much facilitated by more efficient technologies in transferring vectors to peripheral blood T cells. First applied to treat a melanoma patient, *ex vivo* TCR-engineered T cells showed remarkable efficacy and tumor selectivity after adoptive transfer.^33^

These research activities together draw the concept that TILs harbor specificity for the cancer they are isolated from and that the cancer specificity can be transferred to naive T cells by engineering with the respective CAR. Adoptive transfer of such engineered T cells conveys profound anticancer activities. However, several hurdles from the cancer cell site were arising, in particular the frequent downregulation in HLA presentation and/or deficiencies in antigen processing by cancer cells making them invisible to T cells.^34^ Within the following two decades, recombinant TCRs evolved toward synthetic immune receptors by combining antibody and T cell immune modules, which finally accelerated basic immune cell research to rapidly progress to clinical exploration.

## Redirecting T Cells by Synthetic Immune Receptors: The First T-Bodies/Immunoreceptors/Cars

The T cell specificity is defined by the TCR αβ chains, however, the activation is initiated by associated signaling domains, in particular the CD3ζ chain. The molecular cloning of the individual chains of the CD3 complex^35^ was a major milestone in the stepwise evolution of synthetic immune receptors. Three teams, the Weiss, Seed, and Klausner team, independently drew the concept to covalently link the signaling CD3ζ chain with the extracellular part of CD8, CD4, or the IL-2 receptor CD25, respectively, to obtain a chimeric receptor with both a targeting extracellular part and a signaling intracellular moiety. Expressed by engineered Jurkat T leukemic cells or cytotoxic T cells,^36–38^ crosslinking the synthetic receptors by target engagement induced T cell activation indicated by induced Ca^2+^ influx and other activation markers. This was a pioneering step since their work established that antigen-driven crosslinking of CD3ζ is sufficient to initiate a functional response in T cells.

**Figure 2.**
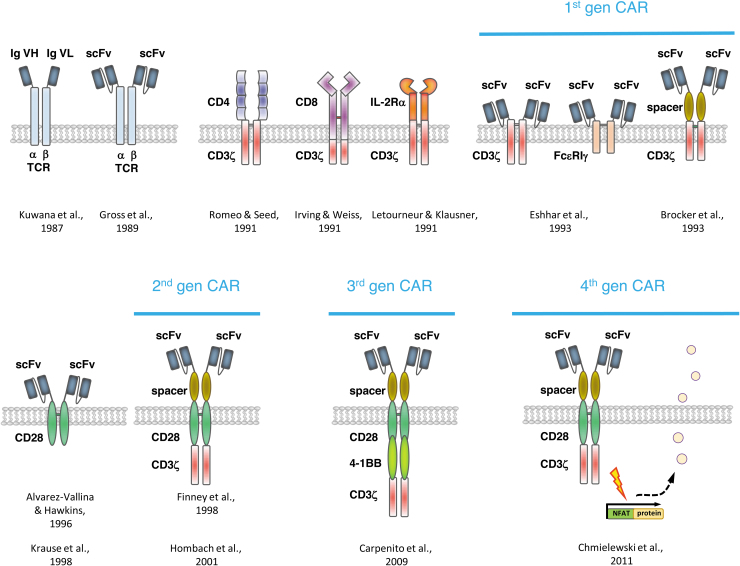
A brief summary of some major steps in the development of CARs. CAR, chimeric antigen receptor.

On the other hand, it remained unresolved how to engraft predefined antigen specificity to a T cell activating complex. Since antibodies are prototypes par excellence to provide antigen specificity, the concept was drawn to covalently link antibody-derived binding domains to the TCR constant regions as first reported by Kuwana *et al.* (Fig. 2).^39^ This was a significant step forward in redirecting T cells toward defined targets since the major histocompatibility complex (MHC) restriction of the TCR was overcome by an antibody.

It was in the late 1980s when the Eshhar's group (Weizmann Institute of Science, Rehovot, Israel) coupled a single-chain fragment of variable region (scFv) antibody specific for the hapten anti-trinitrophenyl to the α- or β-chain of a TCR (Fig. 2).^40^ Such hybrid molecules formed heterodimers with the TCR chains to recruit the T cell activation signaling machinery. To form a functional antibody, the variable region of the immunoglobulin heavy chain was covalently linked by a short flexible peptide to the variable region of the light chain; the scFv antibody format is still commonly used as antigen-sensing domain of recombinant immune receptors. The Eshhar's group also linked the scFv to the extracellular end of the CD3ζ chain or the high-affinity immunoglobulin E receptor γ chain (FcɛRIγ) to produce T cell activation. Indeed, such antibody/TCR chimera induced IL-2 release and cytolytic activity toward target cells in an MHC-independent fashion.^41,42^ The same research team redirected CTL hybridoma cells to cancer cells by a recombinant receptor that binds by the linked scFv antibody to the epidermal growth factor receptor (Her2/neu, ErbB2) overexpressed by a variety of cancers.^43^

Simultaneously, Brocker *et al.* presented a chimeric receptor composed of a single-chain antibody-binding site that was connected over a short spacer to CD3ζ.^44^ Following the same construction scheme Moritz *et al.* reported in 1994 a chimeric receptor ErbB2 and composed of a scFv, a hinge region as a spacer, and the CD3ζ.^45^ All these examples demonstrated that antigen specificity can be redirected toward a defined antigen by a chimeric receptor consisting of an antibody for targeting and the TCR invariant chain CD3ζ or the γ-chain of the IgE receptor for intracellular activation. Redirected recognition of target cells could not only be achieved in T cell hybridomas but also in peripheral blood T cells of humans and mice.^46–48^ These chimeric receptors were named by the Eshhar's group “T-body” as a hybrid of an antibody and a TCR, subsequently by our and other groups “immunoreceptors” and are now known as “chimeric antigen receptors”, precisely nowadays CARs of first generation.

The fundamental advantage of the strategy is that engineering with a synthetic receptor *ex vivo* creates expandable antigen-specific human T cells circumventing the limitations of active immunization to prime T cells *in vivo*. Another crucial property is that the CAR not only provides MHC-independent targeting but also initiates T cell activation. This is based on the discovery of the CD4 and CD8-p56(lck) complexes and leads to the understanding how receptors that lack intrinsic signaling control the T cell response to antigens by association to nonreceptor kinases (reviewed by reference^49^). Taken together, the concept of a downstream protein tyrosine phosphorylation cascade initiated by the TCR ζ-chain or the immunoglobulin receptor γ-chain was applied to design the first hybrid receptors that convert binding to defined target into cellular activation.

Because such chimeric receptors bind their cognate target independently of MHC presentation, these scFv-containing receptors can basically also recognize nonclassical T cell targets like carbohydrates and lipids. As early as in 1997, our group demonstrated CAR-redirected T cell targeting toward the carbohydrate TAG72,^50^ a cancer-associated antigen highly expressed by gastrointestinal carcinoma cells; other examples of nonprotein targeting followed with time.^51^

A major step toward exploring applicability of CAR T cells for therapeutic application was made in 1999 by Sadelain's group (Memorial Sloan Kettering Cancer Center [MSKCC], New York, NY) by redirecting patients' T cells toward cancer cells through such chimeric receptor *in vitro*.^52^ These CAR T cells mediated substantial cytotoxicity, however, showed only poor capacities to expand upon repetitive antigen engagement. Subsequently, our group at Cologne University (Cologne, Germany) demonstrated that patient's T cells engineered with an anti-CD30 CAR can recognize and eliminate autologous T lymphoma cells isolated from patient's biopsy.^53^

One of the first demonstrations of *in vivo* efficacy of engineered CAR T cells was provided by Haynes *et al.*^54^ who showed that adoptive transfer of genetically retargeted mouse T cells eradicated transplanted colon carcinomas in mice. However, early transgenic mouse models also revealed that such ζ-chain CARs could not sufficiently activate resting T cells^44,55^ and CAR T cells could only modestly control tumor growth, produced low amounts of IFN-γ, and rapidly entered anergy.^55,56^

In summary, the first prototype CARs of “first generation” evolved in three substantial stages,

(i) the CD3ζ chain, which lacks extracellular domain, was linked to CD8, CD4, or CD25 to allow signal transmission and T cell activation upon crosslinking^36–38^;
(ii) linking an scFv in the extracellular part to CD3ζ or FcɛRI γ^41,44^ made redirecting engineered cells with any predefined specificity possible;
(iii) by retroviral transduction procedures, the fusion receptors were engrafted on the surface of human naive blood T cells paving the way to adoptive cell therapy.^57,58^

The short-lived CAR-driven T cell activation and insufficient antitumor activity stimulated research toward the next step in the evolution of CARs, the “second generation” CAR, to provide sufficient and lasting activation to the engineered T cell.

## A Major Step Toward Clinical Efficacy: Combining Costimulation with the Primary TCR Signal in ine Chimeric Receptor Improves Car T Cell Antitumor Response

TCR signaling controls T cell differentiation^59^ and is physiologically amplified and modulated by a series of concomitant costimulatory signals. TCR engagement of MHC-loaded antigen presented by dendritic cells along with a costimulatory signal induces clonal expansion and differentiation into effector and memory T cells. According to the “two-signal” hypothesis,^60^ the primary TCR signal (signal-1) together with costimulation (signal-2) is required for full activation of naive T cells, to drive amplification, to prevent unresponsiveness (“anergy”), some forms of activation-induced cell death (AICD), and exhaustion. While signal-1 and signal-2 are sufficient for inducing CD8^+^ T cells to proliferate und to produce cytokines, a third signal, signal-3 according to Lafferty's concept,^61^ is required to execute cytotoxic effector functions.^62^

Upon engagement of antigen through the TCR αβ chains, the prototype costimulatory receptor CD28 is recruited to the immunological synapse to stabilize the TCR microcluster and enhance TCR signaling. This process results in increased cytokine production, cell cycle progression and amplification, differentiation and survival, and in altered epigenetic structure and cellular metabolism. Other costimulatory receptors, such as 4-1BB (CD137), CD27, OX40, and ICOS, are also involved, each addressing a different, however overlapping panel of functions.^63^ Based on the understanding of the relevance of costimulation in mediating sufficient T cell activation, it was disputed how to provide signal-2 in addition to primary T cell signaling in the absence of APCs. The prototype CD3ζ CAR, as it was in use in the late 1990s, was by itself not capable to provide costimulatory help.

From the historical perspective, cytokine gene transfer was one of the first strategies to enhance T cell function by providing additional help. In particular, engineering with the IL-2 cDNA allowed robust amplification of T cells in the long term.^64,65^ The development was dropped, however, since IL-2- or TNF-α-modified TILs did not show clinical benefit over nonmodified TILs.^66^ Evidence reported by the Sadelain group^52^ indicated that IFN-γ and IL-2 release upon redirected activation of patients' T cells was increased when the targeted cancer cells express CD80, the ligand for CD28 on T cells. To provide a costimulatory signal for robust signaling, Alvarez-Vallina and Hawkins^67^ and subsequently the Sadelain group^51^ linked an extracellular scFv for antigen engagement to the transmembrane and signaling domain of CD28 resulting in a scFv-CD28 receptor (Fig. 2). Such chimeric costimulatory receptor induced IL-2 release and prevented AICD in engineered T cells. In summary, the scFv-CD28 receptor demonstrated that costimulation can be provided to engineered T cells in an antigen-triggered fashion in the absence of CD80 on the tumor cell and in the absence of APCs.

Consequently, several groups tried to combine antigen-dependent CD3ζ signaling by the CAR and concomitant CD28 costimulation within one receptor molecule to augment T cell activation.

It was Finney *et al.*^68^ who demonstrated that integrating the CD28 endodomain into the signaling chain of the prototype ζ-chain CAR in a membrane proximal position resulted in a CD28-CD3ζ dual signaling receptor capable of inducing IL-2 release in engineered Jurkat cells. Our group first demonstrated in 2001 that human blood T cells can fully be activated by a CD28-ζ CAR independently of CD80/CD86 on cancer cells indicating CAR-autonomous signaling through both CD3ζ and CD28.^69,70^ The demonstrated full CAR-driven activation of primary blood T cells was a crucial step toward clinical application; T cell hybridoma or leukemic cells used before are not adequate in this respect due to their neoplastic transformation.

Both CD28 and CD3ζ signaling were simultaneously required to provide a receptor-triggered, MHC-independent fashion in human T cells,^69,70^ once more sustaining the concept to integrate CD28 and TCR CD3ζ-chain into one dual-signaling receptor. Shortly afterward, the Sadelain group also showed the benefit of integrating CD28 signaling domain into the CD3ζ receptor to provide full T cell activation.^51,71^ This dual-signaling CAR was capable of redirecting patient's blood T cells toward autologous cancer cells as shown by our group using carcinoembryonic antigen-specific CAR T cells redirected against autologous colon cancer cells isolated from a biopsy^72^; other examples for leukemia and solid cancer followed from other groups.^73,74^

The CAR with integrated costimulatory and primary signaling domain is now defined as CAR of “second generation” (Fig. 2); any other costimulatory domain, each with some different impact on T cell function, can be added to the primary signaling domain, including 4-1BB (CD137) as reported in 2004,^75^ CD27, OX40, or ICOS.^76–79^ Basically, the second-generation CARs provide the T cell the adequate signals required to bypass anergy and apoptosis, thereby extending CAR T cell function toward more potent antitumor activities, increased cytokine production, and improved amplification and persistence compared with first-generation CARs.^75,76,80,81^ The benefit in extending T cell performance became obvious when metastases in mice were more efficiently reduced by CD28-ζ CAR T cells compared with the previously used ζ CAR T cells of first generation.^81^ In a parallel development based on the rationale that lymphocyte-specific protein tyrosine kinase (Lck) promotes CD3ζ immunoreceptor tyrosine-based activation motif phosphorylation, the src-family kinase Lck signaling domain was integrated into the ζ-chain CAR, also resulting in augmented T cell activation.^82^ This type of CAR has not been studied intensively in the following years, however, nicely demonstrates the modularity and flexibility of CAR design.

Given the biological differences in costimulation by the various receptors, comparative analyses of the CD28-ζ CAR versus 4-1BB-ζ CAR expectedly confirmed that the individual costimulatory domains differently impact a number of functional properties, including the metabolism,^83^ T cell memory development,^83,84^ and antigen-independent tonic signaling.^85^ CD28-ζ CARs induce a strong and rapid T cell activation while 4-1BB-ζ CARs gradually induce the T cell response that persists over a longer period. Moreover, the activation-induced cytokine profile of CAR T cells depends on CAR-provided costimulation; for example, IL-2 and IL-10 are released predominantly upon CD28-ζ and less by 4-1BB-ζ CAR signaling.^86^ Debulking larger tumor mass is more rapidly performed by CD28-ζ CAR T cells than 4-1BB-ζ CAR T cells, however, the latter compensate the lower cytolytic activity by prolonged activities in time.^87,88^ It is also becoming clear that the sensitivity to tumor-driven repression is different between the second-generation CARs; CD28-ζ CAR T cells are resistant to transforming growth factor (TGF)-β repression while 4-1BB-ζ CAR T cells are efficiently repressed, which is crucial when targeting solid cancer lesions with high TGF-β load.^89^

While each costimulatory signal triggers a distinct, although overlapping pattern of T cell responses, for adoptive cell therapy the combination of two costimulatory signals was hypothesized to be beneficial.^90,91^ First, the group by Carl H. June (University of Pennsylvania, Philadelphia, PA) showed in 2009 that CAR T cells with both CD28 and 4-1BB costimulation successfully controlled large established tumors, which was not the case for CARs providing one costimulatory signal only.^92^ The observations were confirmed by the Sadelain group^91^ demonstrating a combined activation of the phosphoinositide 3-kinase/protein kinase B (PKB, AKT) pathway through combined CD28-4-1BB-ζ CAR signaling. Engineered CAR T cells showed increased Bcl-xL, decreased apoptosis *in vitro*, and augmented tumor elimination in a mouse tumor model. The CAR takes benefit of the cooperativity of CD28 and 4-1BB in augmenting antitumor activity by inducing lytic effector molecules and polarizing toward a type-1 cytokine response. This type of CARs with two costimulatory domains in any combination was established as “third generation” CARs (Fig. 2).

Later, other combinations of costimulatory signals came to interest in specific situations, including the combination CD28-OX40^93^ which showed superior activity in T cells in advanced stages of development.^94^ The CD28-OX40 CAR was shown to repress CD28-induced IL-10 release that compromises T cell activity.^95^ ICOS together with CD28 or 4-1BB costimulation increased the CAR T cell persistence *in vivo*; MyD88/CD40 costimulation improved *in vivo* amplification.^96^ Expanding research in third-generation CARs over the following years, however, revealed that the combination of two costimuli is not in every cell type potent to augment the antitumor efficacy^95^; current research is focusing back on second-generation CAR T cells, particularly since, in the meantime, these CARs have produced spectacular success in the treatment of hematologic malignancies.

## CARs of Second Generation are Taking the Front Seat in Clinical Exploration

Primarily developed to understand TCR-mediated activation, chimeric receptor-redirected T cells rapidly developed toward clinical application bringing hope to control tumors in the long term. First-generation CARs were explored in the early trials targeting ovarian carcinoma in 2006,^97^ metastatic renal cell carcinoma,^98^ and neuroblastoma^99^; however, CAR T cells insufficiently persisted in a functional state to mediate lasting antitumor efficacy.^97,100^ It is rather the loss of function than the lack of persistence of first-generation CAR T cells that basically contributes to the treatment failure since even a decade after application CAR T cells could be molecularly detected in patients treated for HIV infection.^101^

For further clinical exploration, CARs of second generation targeting CD19 came into focus to treat B cell malignancies. Using CD19 as target has several advantages; first, CD19 is a B lineage-associated antigen expressed by most B cell leukemia and lymphoma, second, hematologic malignancies are more easily accessible than solid cancer cells, and third, CD19 CAR T cell-induced aplasia of healthy B cells can be clinically managed.

The first successful treatment of a patient with chronic lymphocytic leukemia with CD19-specific CAR T cells was reported in 2011 by June's team at the Abramson Family Cancer Research Institute at the University of Pennsylvania.^102^ In trials simultaneously performed at the National Cancer Institute (NIH/NCI) and Memorial Sloan-Kettering Cancer Center (MSKCC), both using CD28-ζ CAR T cells, and at the Abramson Center/University of Pennsylvania, using 4-1BB-ζ CAR T cells, all achieved spectacular treatment results of relapsed/refractory B cell malignancies. At the Abramson Center/UPenn and MSKCC, anti-CD19 CAR T cells produced complete remissions in 60–93% of B cell acute lymphocytic leukemia (B-ALL) and 50–93% overall responses in the treatment of B cell non-Hodgkin's lymphoma.^103–108^ In contrast to first-generation CARs, second-generation CAR T cells amplified *in vivo* and persisted for prolonged time in the peripheral blood upon application to lymphoma patients.^109^ While the first treatment responses were very similar, the respective CARs differ in their costimulatory domains with some differences in the response kinetics and pharmacology.^110^

All trials followed the same clinical regimen primarily developed in the late 1980s by the Rosenberg team at the NCI, comprising leukapheresis to obtain patient's T cells, *ex vivo* engineering of T cells with a CAR-encoding retro- or lentivirus, amplification of T cells to clinically relevant numbers, and patient “preconditioning” by nonmyeloablative lymphodepletion before CAR T cell infusion. However, due to a number of variables during the entire process on the clinical as well as on the manufacturing side, there is currently no consensus protocol available, which makes comparisons between clinical trial data difficult. Apart thereof, all trials achieved dramatic complete remissions of the targeted disease, some are long lasting. Based on these and more upcoming spectacular results, the journal “Science” proclaimed the CAR T cell therapy together with the checkpoint blockade as “breakthrough of the year 2013.”^111^

As early as in 2014, several CAR T cell products targeting CD19 received U.S. FDA “breakthrough designation” like Memorial Sloan Kettering Cancer Center CD28-ζ CAR for adult B-ALL,^112–114^ University of Pennsylvania 4-1BB-ζ CAR for pediatric ALL,^115^ and National Cancer Institute/Kite CD28-ζ CAR for diffuse large B cell lymphoma (DLBCL)^116^ (NCT00924326). In 2015 Kite's CD28-ζ CAR (NCT02348216) and in 2016 Juno's 4-1BB-ζ CAR (NCT02631044) followed for the treatment of DLBCL.^117^

Based on this and other data, in August 2017 U.S. FDA approved Novartis' 4-1BB-ζ CAR-based product tisagenlecleucel (CTL-019; Kymriah^®^) for pediatric B-ALL^118–121^ and in May 2018 for DLBLCL. CD28-ζ CAR-based Kite Pharma/Gilead axicabtagene ciloleucel (axi-cel; Yescarta^®^) was approved in October 2017,^122–124^ followed by CD28-ζ CAR-based brexucabtagene autoleucel (KTE-X19; Tecartus^®^)^125–127^ in 2020, and in February 2021 Juno Therapeutics' 4-1BB-ζ CAR-based lisocabtagene maraleucel (liso-cel; Breyanzi^®^).^128^ These CAR T cell products are the best clinically studied CARs to date and are being applied for the treatment of B cell malignancies in the standard-of-care setting. In March 2021, U.S. FDA approved Celgene/BMs' anti-BCMA CAR Abecma^®^ (idecabtagene vicleucel) for the treatment of multiple myeloma. Taken together, the emerging field of CAR T cells targeting CD19 has become a paradigm for evaluating CAR T cell therapy (reviewed by reference^76^).

With the first trials, it also became clear that CAR T cell administration is often associated with severe systemic toxicities that require intensive care, including the cytokine release syndrome (CRS) or the poorly understood immune effector cell-associated neurotoxicity syndrome (ICANS), which are both usually transient and reversible. Nowadays, clinical scoring and handling protocols of side effects are becoming more established making clinical management more standardized. Research is currently aiming to improve safety in this respect by fine-tuning the CAR or T cell function to reduce the risk for CRS, for instance by disrupting the granulocyte/macrophage colony-stimulating factor locus^129^ and by cytokine sequestering by a nonsignaling IL-6 receptor.^130^

The numbers of active trials are steadily increasing in the United States, China,^131^ Europe,^132^ and other countries establishing CAR T cell therapy in the forefront of treatment modalities. However, evaluating clinical data in a comparative fashion is complex since a broad diversity in the manufacturing processes and in clinical protocols exists. To address the situation, the CARAMBA consortium set up a first consensus procedure for a trial with 10 partners from 6 EU countries targeting SLAMF7-specific CAR T cells, manufactured by transposon-mediated gene transfer, to treat patients with multiple myeloma (NCT 04499339).

Manufacturing the respective cell products for clinical application was first performed at academic centers, and then taken by commercial suppliers; however, CAR T cell manufacturing for clinical trials remains a bottleneck. Technically, T cells are engineered from the patient's leukapheresis product by a viral vector and amplified in a local or centralized good manufacturing procedure (GMP)-certified facility before given back to the patient.^133^ While γ-retroviral and lentiviral vectors are used from the beginning, now nonviral vector systems like sleeping beauty transposon and, first in 2017, CRISPR/Cas genome editing^134^ are also used. The alternatives to viral gene transfer technologies are steadily increasing, including extrachromosomal nonintegrating S/MART vectors, as recently reported for CAR T cell engineering *in vitro*.^135^ The manufacturing process was initially, and still is in the majority of trials, done by a manual process^136^; the entire procedure currently requires 12–15 days; modifications to shorten the process down to 10 days and less are explored in pilot trials.

In parallel to the stepwise optimization of CARs, biotech companies started to develop automated, supervised, and closed manufacturing systems aiming for local use in the hospital to produce the cell product in the near vicinity of the patient. Such decentralized manufacturing spots, however, still require tremendous efforts in investments with respect to the clean room facility, training the manufacturing team, setup of quality control, and other mechanisms to allow GMP-compliant manufacturing of the CAR T cell product.^137^ Despite these hurdles, the number of decentralized manufacturing spots is continuously growing establishing a new biotech infrastructure on the hospital campus. This is a fundamentally new development beyond the classical hospital pharmacy with respect to producing an individualized living drug for a specific patient by genetic engineering and biotechnological manufacturing.

There are a number of variables that affect the quality of the final CAR T cell product, and not all are identified yet. Among these variables are the ratio of CD4^+^/CD8^+^ T cells, the expression level of the respective CAR, the exhaustion and terminal differentiation level of the engineered T cells, and others.^138^ Research on the preclinical as well as clinical side is aiming to identify and standardize crucial parameters to deliver a potent cell product in a robust process and in due time. It is currently under investigation whether a specific T cell subset such as memory stem cells, a specific polarization of T cells before and/or after genetic engineering, and during expansion will provide clinical benefit. The complexity of CAR design becomes exemplarily obvious by the fact that different anti-BCMA CARs with similar affinity drive T cell activation differently^139^; targeting the same epitope of CD19 with similar affinity, however, by a structurally different CAR, results in different degrees of T cell activation.^140^

Along with the success of the first clinical trials in the first decade, established companies as well as a number of young companies started to develop their own CAR products. Companies are running target discovery platforms to identify new and more suitable targets for CAR T cell therapies of particular malignancies; the field is now more than rapidly growing worldwide. While successful in the treatment of hematologic malignancies, the development of CAR T cell therapies for treating solid tumors is recognized to require more sophisticated strategies to overcome the broad panel of tumor-defense mechanisms, including the physical barrier to enter the tumor tissue, the immunosuppressive stroma, the antigen loss or downregulation, and others.^141,142^

## Trucks: Car T Cells Become “Living Factories”

While B cell malignancies are being successfully treated, solid tumors so far resisted CAR T cell treatment that is thought to be due to the specific tumor stroma, including physical barriers in penetrating the tumor tissue, active immune repression, and hostile environment within the tumor and others.^143^ To address the situation, researchers are aiming to use the CAR T cells not only as targeted “living drugs” but also to produce a transgenic therapeutic protein to sustain the CAR T cell attack. Such “fourth-generation” CAR T cells, or TRUCKs (“T cells redirected for universal cytokine-mediated killing”) as described by our group, produce a therapeutic protein, mostly a cytokine, under control of a CAR-inducible promoter (Fig. 2). The protein is only produced and released when the CAR T cell is engaging the cognate target, thereby delivering the “payload” at the targeted tumor site and only there.^144,145^ In tissues without CAR-recognized target, no payload protein is released. CAR engagement of antigen triggers a downstream signaling cascade that drives by binding to a nuclear factor of activated T cells responsible enhancer element a minimal promoter for synthesis of the transgenic protein, going far beyond cancer cell targeting. The concept is universal, a number of proteins can basically be delivered; for instance, the proinflammatory IL-12, induced and constitutively expressed,^146–148^ acts in trans for attracting and activating innate cells in the targeted tumor lesion, repolarizes tumor-resident macrophages and resist inhibitory elements in the tumor tissue, including Treg cells and myeloid-derived suppressor cells.^149^

Combining the CAR T cell attack with an innate immune response may represent a strategy to prevent tumor relapse by remaining cancer cells that downregulated the target antigen and are invisible to CAR T cells. CAR T cells targeting vascular endothelial growth factor receptor-2 and releasing inducible transgenic IL-12 produce augmented tumor regression.^150^ TRUCKs with inducible IL-12 and targeting Epstein/Barr nuclear antigen (EBNA)-3C responded even more effectively; released IL-12 recruited additional immune cells, which are generally missing in proximity of lymphoproliferation in immunocompromised posttransplantation lymphoproliferative disorder patients demonstrating a strategy how healthy immune cells can be mobilized to control EBV-associated lymphoproliferation.^151^ These and other examples show a further development of CAR T cells from “living drugs” toward “living factories” that produce therapeutic proteins with several advantages: (i) the therapeutic protein is deposited in the CAR-targeted lesion, which is otherwise not accessible, (ii) inducible release avoids systemic toxicity of the therapeutic protein while accumulating in the targeted tissue, and (iii) the protein is continuously released achieving locally high levels as long as the TRUCK cell is activated.

In a first-in-man trial, researchers modified TILs, which are thought to be tumor specific without the need to be redirected by a CAR, with inducible IL-12; such IL-12 TILs were highly efficacious in the treatment of melanoma since a therapeutic dose was achieved with 50- to 100-fold fewer cell numbers than commonly with unmodified TILs.^152^ However, IL-12 TIL therapy was accompanied by severe systemic toxicity, most likely to leakage of the vector in highly activated TILs.

Like cytokines, TRUCK cells were engineered releasing an antibody to block PD1,^153^ a proteolytic enzyme to degrade the tumor matrix,^154^ or the costimulatory ligand CD40L to prolong T cell activation,^155,156^ or the combination of CCL19 and IL-7 to recruit endogenous immune cells and antitumor memory^157^; the list of transgenic payloads is continuously growing.^158,159^

A significant further development was made by enhancing T cell intrinsic functions by the inducible release of an immune response modifier. We first demonstrated the feasibility of the strategy by engineering a TRUCK with inducible release of IL-18 that alters the expression of two key transcription factors, T-bet (Tbx21) and forkhead box protein-O1 (FoxO1), in CAR T cells. IL-18 was identified to tip the balance toward improved granzyme-mediated killing capacities.^160,161^ IL-18 augmented IFN-γ secretion and proliferation of T cells activated by the endogenous TCR^159^ and CAR.^160,161^ TRUCKs with inducible IL-18 reinforce their own killing capacity when engaging their cognate target, thereby capable to control larger tumors than the corresponding CAR T cells.^162^ Another example of an action in cis is the release of IL-15 to polarize CAR T cell function.^163^ The path for using “living factories” for therapeutic purposes is opened; other possible applications for cancer therapy and beyond will likely be envisaged in the near future.

During three decades of development, CAR T cell engineering has steadily followed an evolution to improve T cell potency and sustainability after adoptive transfer producing an increasing number of CAR generations and strategies to use engineered cells as factories. The evolution is clearly driven by the modularity of the major components of a CAR; the impact of each individual domain on CAR T cell performance is nowadays recognized more important than previously anticipated. Research in this field is becoming much diverse and produced different CAR candidates with different advantages; for details, I refer to a recent review.^164^ Two developments are currently triggering the next steps in CAR evolution: spiking a “universal” CAR with different targeting domains and engineering logic gating CARs as discussed below.

## Cars Evolve Toward Universal Adaptor Receptors And Logic Gating Devices Controlling Cellular Functions in Space and Time

Multiple receptors with different binding partners can be used to transmit activating or inhibiting signals to differentially program T cell activation or inhibition.^165^ While the approach shows the power of synthetic receptor combinations, however, there is an increasing complexity in the multicomponent systems that require optimization of each component with respect to multiple parameters questioning a robust therapeutic use. To address the shift of targetable antigens within a progressing tumor, CARs are understood as transmembrane signaling molecules with tunable specificity by noncovalently spiking with an adaptor protein that confers the targeting specificity; adding different adaptor proteins allows targeting multiple antigens by the same CAR. The strategy has the advantage of flexibility in specificity to address the risk of antigen escape by administering different targeting antibodies to the patient. In addition, the concepts allow an ON/OFF switch by adding or withdrawing the biotinylated antibody mitigating toxicity. As a proof-of-concept, Urbanska *et al.*^166^ designed a CAR with a biotin-binding domain to allow binding of any biotinylated antibody for target recognition. Based thereon, several other adapter-protein-spiked CARs were developed in the following years.^167–174^ Some of these “uniCARs” are in clinical trials^175^ to explore the benefits in targeting antigen-heterogeneous tumors.

Recent developments understand conventional CARs to produce single-input, single-output signaling, which is thought not to be sufficient to address the complexity of antigen pattern for specific cancer cell targeting. To solve the situation, “AND” and “OR” Boolean logic gating is currently discussed and translated into a CAR design.

“OR” gating CARs harbor two binding domains, for example, an anti-CD19 and anti-CD20 scFv, both covalently linked together in a specific order.^176^ Binding of either cognate antigen is sufficient to induce CAR-redirected T cell activation. Such CD20-CD19 or CD19-CD22 dual specific CAR T cells and other combinations are currently in clinical exploration^177^ with the aim to prevent tumor relapse by cancer cell variants that lost one of the targeted antigens.

In comparison, “AND” gating CARs turned out to be technically more challenging.^178^ The primary CD3ζ signal was separated from the costimulatory signal, each CAR providing only one signal and each CAR recognizing a different antigen on cancer cells; full T cell activation is only provided upon simultaneous engagement of both targets.^179,180^ Some leakage in signaling of the CAR with signal-1, however, weakens logic gating of such dual CAR T cells. In a further development, Ho *et al.*^181^ proposed to deliver upon engagement of one antigen, a conditionally active molecule that becomes cytotoxic only upon recognition of the second antigen.

As an alternative strategy, two groups simultaneously proposed to take advantage of the synthetic Notch receptor (synNotch) system to achieve combinatorial antigen recognition and to control T cell recognition of complex targets.^182,183^ The CAR-like synNotch receptor induces the transcriptional activation of an authentic CAR through proteolysis mediated by the transmembrane core domain; the authentic CAR recognizes a second antigen on the cancer cell for driving T cell activation. The strategy has the advantage to cause less off-tumor toxicities compared with a conventional CAR.^184^ The synNotch receptor harbors an scFv for target recognition, as well as Gal4-VP64 or TetR-VP64, which are transcriptional effector domains required to induce CAR expression. This allows T cell activation only when both the synNotch CAR and the authentic CAR engage their respective targets. The synNotch system is a strategy to integrate multiple cellular inputs into predefined signaling cascades to trigger specific cellular functions in space and time. This will open new fields how to control cellular functions dependent on the environmental tissue. As a result of this development, the discussion arises how to make CAR T cells not only targeting defined tissues but also sensitizing and modulating the tissue they are penetrating.

## Toward Environment Sensing Car T Cells

During the last years, identification of the hallmarks of the tumor microenvironment (reviewed by references^185,186^) have driven research toward a CAR design that allows modulating the tumor stroma. The strategies include (i) enabling CAR T cells to home to and to infiltrate the tumor tissue, (ii) disrupting the suppressive signaling axes in the tumor, (iii) inducing autocrine stimulation to augment CAR T cell amplification and persistence, and (iv) inducing an endogenous immune response.

To home to the tumor site, CAR T cells were guided by chemokine receptors, like CCR8, CXCR6, and others.^187–189^ It has not only improved homing to the tumor tissue but also attacking the tumor stroma that augments antitumor efficacy. As a result of these analyses, the concept is drawn that successful eradication of advanced tumors requires CAR T cells that target the stroma in addition to targeting the specific cancer cells.^190,191^ The issue is still underestimated and requires to envision tumors not only as accumulated cancer cells but as an organ orchestrating cancer cells within a connective and immunosuppressive tissue.

Once infiltrated into the tumor tissue, CAR T cells need to stay activated in the hostile environment facing a number of inhibitory molecules, including TGF-β, B7H1, mucin, and prostaglandin E_2_ (PGE_2_), a bioactive lipid often upregulated in tumors that activates protein kinase A (PKA).^192^ To counteract repression, several new CAR strategies are being exploited during the last years pointing to a more advanced functional remodeling the T cell response. To counteract PGE_2_, CAR T cells were engineered to express a peptide inhibitor of PKA translocation to the immune synapse that improves CAR T cell activation and augments the antitumor attack.^193^ CAR T cells that express catalase are capable of metabolizing suppressive H_2_O_2_ in the tumor, thereby also augmenting the efficacy of their antitumor attack.^194^

Antibody mediated blockade of immune checkpoints PD1, PD-L1, CTLA-4 and others have revolutionized treatment modalities in the last years^195^ and are currently translated to CAR T cell therapy by combined treatment in trials, for example, CD19-CD22 CAR T cells together with pembrolizumab (anti-PD1 antibody) (NCT03287817), and CD19 CAR T cells together with utomimulab (anti-CD137 antibody) (NCT03704298) are currently explored. Alternatively, the so-called switch receptors are coexpressed that comprise a suppressive factor-binding domain and an intracellular activating domain, thereby converting engagement of a negative factor into positive T cell activation. A first example is a chimeric receptor binding IL-4 through an IL-4 receptor-α chain and transmitting a positive signal through the IL-7 receptor α-chain or the IL-2 receptor β-chain, thereby augmenting the T cell antitumor activity.^196,197^ Others are engaging CTLA-4 or PD-1 while transmitting CD28 costimulation.^198–201^ A switch receptor composed of a binding domain for T cell immunoreceptor with Ig and ITIM domain (TIGIT) protein and a CD28 signaling domain augments cytokine release and T cell activation.^202^ To modify CAR T cell activity, “armored” CARs with transgenic expression of an additional costimulatory protein, for example, 4-1BB-L, were designed by Stephan *et al.*^90^; the CD19 CAR with 4-1BB-L is now explored in a clinical trial (NCT03085173).

Addressing the crucial role of inhibitory receptors, several groups are currently following the strategy to genetically edit the inhibitory receptors in engineered T cells. CRISPR-Cas9-mediated disruption of PD-1 augments killing efficacy of CD19 CAR T cells toward PD-L1 tumors in a mouse model.^203^ As a proof of concept, a recent trial demonstrated feasibility and safety of CRISPR-depleted PD-1 and the endogenous TCR αβ in NY-ESO-1 TCR-engineered T cells.^204^ This is a step forward toward genetically edited T cells that are fine tuned for redirected targeting. One application is making allogeneic CAR T cells clinically applicable by targeting the TCR α-locus and the β2 microglobulin locus and to make them resistant to repression by targeting PD-1 or Fas locus.^205–207^ In continuation of the technology, a pooled knockin screen using CRISPR-Cas9 integration into the TCR α-locus was applied to test for transgenes improving performance of CAR T cells against solid cancer; a newly identified prototype that addresses the issue was a TGF-β engaging 4-1BB signaling switch receptor.^208^

Given the central role in T cell repression, attempts to counteract TGF-β were early undertaken during CAR development; application of TGF-β blocking antibodies augment antitumor activity.^209,210^ T cells equipped with a mutant TGF-β receptor gained superior activity and survival in the presence of TGF-β.^211–213^ Coexpressed with the CAR, a recombinant dominant-negative (DN) receptor sequesters TGF-β from the environment to protect the CAR T cell from repression.^214^ A clinical trial currently explores the benefit of TGF-β DN receptor while targeting prostate cancer (NCT 03089203). In a further development, TGF-β-targeting CARs and TGF-β switch receptors^215,216^ provide a stimulatory signal to the CAR T cell in contrast to the DN-TGF-β receptor. Costimulation through CD28 counteracts suppression by TGF-β,^217^ whereas 4-1BB costimulation does not. TGF-β resistance upon CD28 CAR T cell stimulation is due to released IL-2 and IL-2 receptor signaling. To provide IL-2 receptor signaling in the absence of IL-2, a synthetic receptor was designed that binds IL-7 and provides IL-2 receptor signaling to overcome TGF-β repression.^89^

There are also examples for an emerging concept to sensitize the tumor tissue before a T cell attack. Early studies interfered with the apoptotic pathway of lymphoma or melanoma cells to make them susceptible to CAR T cells.^218,219^ A recent strategy is based on pretreating tumors with senescence-inducing drugs prior application of CAR T cells that recognize urokinase-type plasminogen activator receptor broadly expressed during senescence to target and ablate cancer cells that were turned into senescence.^220^

## TCR-Like Cars and CAR-Like TCRs: The Same Immune Receptor at The End?

There are two challenges when redirecting T cells by a transgenic TCR, the mispairing with the endogenous TCR and the rate-limiting competition with the CD3 signaling complex and downstream kinases. While the latter also apply to CARs, modifications of the TCR chains prevented mispairing of the transgenic with the endogenous TCR; adding a signaling domain to the intracellular chains of the TCR could overcome both limitations to a certain extent,^221^ ending up in a hybrid receptor similar to first-generation CARs.

In contrast to TCRs, the weakness of prototype CARs is still that intracellular proteins cannot be targeted. However, some groups demonstrated that intracellular proteins presented by HLA/peptide complexes can basically be targeted by CAR T cells using a peptide/MHC-specific antibody for binding; this was as exemplarily shown for targeting NY-ESO1.^222^ To specifically target EBV-infected B cells, a TCR-like CAR was recently reported that recognizes an EBNA-3C-derived peptide in HLA-B*35 in a TCR-like fashion.^151^ Such TCR-like CAR T cells may have extended capacities compared with classical CARs or TCRs in that intracellular targets can be recognized without major competition with the native TCR or CD3. This may become of major interest in the near future when targeting HLA-presented proteins with tumor-specific mutations being explored on a more systematic and broader basis.

## We Just Started to Learn

Looking at the history of cancer therapy, it is rare that a single agent achieves remission of advanced tumors in the long term. Although extraordinarily efficacious in the treatment of hematologic malignancies, CAR T cell therapy will become one pillar in the orchestra of treatment modalities in oncology. During the last three decades, much research has been done on the CAR design to improve antitumor efficacy leading to a growing family of CAR generations. By multilayered approaches, the critical needs of T cells as “living drugs” are being identified and stepwise addressed. Basic research has followed a clear line of improvements *in vitro*, in mouse models and subsequently translated the CAR T cell strategy in clinical exploration. The success of the second-generation CAR in clinical trials justifies to continue our efforts in making CAR T cell therapy efficacious in targeting solid cancer and in exploring CAR T cells for the treatment of various other diseases.

Currently, much effort is invested to optimize the individual CAR domains on a case-to-case basis with the aim to obtain clinically efficacious CAR T cell products. A growing number of factors are being identified that affect CAR activity, including the targeted antigen, its epitope, its density, and mobility on the cell membrane, the CAR-binding affinity and CAR spacer length, the clustering on T cell surface, and the cell interacting avidity, among others. Consensus on a “one-for-all” prototype CAR design is not in sight, and probably will not be achievable; currently thorough optimization still needs to be performed for each CAR on an empiric basis to obtain a synergistically complemented, modularly calibrated, and clinically efficacious CAR T cell product. Despite extensive safety measures and ongoing optimization in the molecular design, CAR T cell therapy remains experimental with the risk of unpredictable side effects; careful clinical exploration is and will be needed to make such powerful immunotherapy robust and safe.

There was a long way from early recombinant TCR and T-body engineering toward the first commercial approval of a form of gene transfer therapy by the U.S. FDA and European EMA. The success of the anti-CD19 CAR T cell therapy so far clearly indicates the power of immune cell therapy to control leukemia/lymphoma transforming the clinical management of these diseases. CAR T cell therapy demonstrates greater incremental effectiveness and similar cost-effectiveness compared with prior U.S. FDA-approved pharmacological innovations.^223^ However, great challenges in translating CAR T cell therapy to clinical application remain, including the paucity of preclinical models to evaluate safety and efficacy.

Adoptive cell therapy is now becoming a pillar in immuno-oncology: TILs with or without checkpoint blockers for the therapy of tumors with high mutational burden, TCR, or CAR engineered T cells for tumors with low mutational burden or targetable tumor-related antigen. Efforts are made to engineer T cells, NK cells, and TILs with CARs with greater precision. The field is rapidly moving forward; multiple novel receptors are making preclinical impact, and many biotech and pharma companies are preparing for the next generation of clinical trials.

There are still many nonunderstood hurdles that hamper translating the therapy in particular to solid cancer. There is consensus that CAR T cells require more fine tuning and the immune and tissue environment needs additionally to be addressed. With respect to engineering, recent advancements in genetic editing and in designing novel functional circuits enable us to augment T cell fitness and to design “intelligent” CAR T cells. Recognizing redirected T cells as “living factories,” we can produce a therapeutic protein on demand and in a predefined tissue, which opens new concepts to fight cancer. Technical considerations remain with respect to manufacturing practicability, clinical trial approaches, cell quality and persistence, and patient management. Recent advances also fuel hope that synthetic immunology, genetic engineering and editing, and cell manufacturing will broaden CAR-redirected cell therapy to the relevant T cell subset and to other immune cell types, and to develop new applications beyond oncology, including autoimmunity and infectious diseases.
